# Psychosocial difficulties as a predictor of overall functioning in individuals with schizophrenia: a case-control study

**DOI:** 10.1192/j.eurpsy.2024.1571

**Published:** 2024-08-27

**Authors:** L. Mustoo Başer, O. Aydɪn

**Affiliations:** Psychology, International University of Sarajevo (IUS), Sarajevo, Bosnia and Herzegovina

## Abstract

**Introduction:**

Difficulties in emotional, cognitive, behavioral, and social functions are considered important factors of global functioning in schizophrenia.

**Objectives:**

A better understanding of difficulties in the mentioned facets might lead to the development of better-adjusted treatment approaches for individuals with schizophrenia, as well as impacting to reduction and elimination of stigma in Bosnia and Herzegovina’s wider social context in relation to difficulties spanning the daily life of individuals with schizophrenia.

**Methods:**

Thirty- one patients with schizophrenia (SCH), and 30 healthy controls (HC) participated in our study. The Adult Self-Report (ASR), Achenbach’s scale for adults (ASEBA- Achenbach System of Empirically Based Assessment), and World Health Organization, Disability Assessment Schedule 2.0 (WHODAS 2.0) tests were administered.

**Results:**

Groups differed in age, education, employment status, marital status, friendships existence, and disability existence. According to the regression equations, thought problems predicted Getting around; withdrawn predicted Getting along with people and Life activities- Household, School/Work in the group of individuals with schizophrenia.

**Conclusions:**

Our study revealed the role of different facets of difficulties in the prediction of global functioning in SCH. These findings might directly point to the importance of eliminating stigmatizing beliefs in a wider social context, developing techniques for improving the social support segment, focusing on healthy family functioning, as well as investigating job presence and perceived quality of life.

**Disclosure of Interest:**

None Declared

